# Transcultural Adaptation of the Breast Cancer Awareness Measure in a Rural Border Area of Greece

**DOI:** 10.7759/cureus.61396

**Published:** 2024-05-30

**Authors:** Panayiota Papasozomenou, Panagiotis Eskitzis, Eleftherios Panteris, Angeliki Gerede, Anastasia Patsiatzi, Menelaos Zafrakas

**Affiliations:** 1 Department of Midwifery, School of Health Science, International Hellenic University, Thessaloniki, GRC; 2 Department of Midwifery, School of Health Science, University of Western Macedonia, Ptolemais, GRC; 3 Department of Obstetrics and Gynecology, Medical School, University of Thrace, Alexandroupolis, GRC; 4 Department of Obstetrics and Gynecology, Medical School, Aristotle University of Thessaloniki, Thessaloniki, GRC

**Keywords:** rural area, breast disease, breast cancer signs, breast cancer, breast awareness

## Abstract

Background: Breast cancer awareness is a concept suggesting that awareness of signs and symptoms and early seeking of medical advice may decrease mortality, especially in limited resource settings.

Methods: A modified questionnaire based on the breast cancer awareness measure (BCAM) by Cancer Research UK was translated into Greek and used for the first time. Participants were women residing in a rural border area in Greece. For statistical analysis the χ^2 ^goodness-of-fit and Cramer’s V test for categorical comparisons were used and Cronbach’s alpha for reliability analysis.

Results: In total, 110 women filled out and returned the questionnaire. Respondents appeared to be inappropriately informed regarding the less common warning signs of breast cancer, the most common age of breast cancer occurrence, the national screening program, and the less important risk factors of breast cancer. On the other hand, most women appeared to be confident in recognizing breast changes and seeking medical advice if needed.

Conclusions: The translated modified BCAM tool can be used to evaluate breast cancer awareness in Greek women. Future campaigns developed by policymakers should focus on improving breast cancer awareness, especially in socioeconomically deprived areas.

## Introduction

Breast cancer is the most commonly diagnosed cancer and the leading cause of cancer death in women in the world, followed by lung and colorectal cancer for both cancer incidence and mortality [1]. Breast cancer accounts for almost one in four cancer cases and one in six cancer deaths in women worldwide, with an estimated 2.3 million new cases and 666,000 deaths in 2022 [1]. In many high‐income countries, mortality rates have decreased since the late 1980s and early 1990s [1-3]. This progress is attributed to earlier diagnosis through mammography screening and increased breast awareness, together with improvements in treatment [2]. However, under certain circumstances, such as in rural and geographically remote areas in high-income countries and in low-income countries in general, mammography screening is neither cost‐effective nor feasible; in such limited-resource settings, the focus is on early diagnosis by ensuring prompt and effective diagnosis and treatment of women with symptomatic lesions [1].

Cancer awareness and breast cancer awareness in particular is a concept that has been advocated in the United Kingdom since the 1990s, suggesting that awareness of signs and symptoms of cancer and early seeking of medical advice may lead to a decrease in disease-specific mortality [4-6]. The most common symptoms of breast cancer include a lump in the breast and/or the axilla, nipple discharge, changes in the nipple-areola complex and/or the skin of the breast, and less commonly changes in the size and shape of the breast and breast pain. Awareness of these symptoms may lead to early detection or at least limit unnecessary delays in diagnosis and treatment. The aim of the present study was to evaluate the knowledge of women in a rural border area in Greece on breast cancer awareness to improve breast cancer prevention services and disease prevention policies, especially in geographically remote rural areas.

## Materials and methods

A modified questionnaire was used, based on breast cancer awareness measure (BCAM), developed by Cancer Research United Kingdom [7], and translated into the Greek language (Appendix 1). The questionnaire was handed out to women attending the educational events for the general public in the frame of the “HEALTH INFO” Project [8]. These educational events were organized by the International Hellenic University in co-operation with local municipal authorities; the general public was informed through local newspapers, local radio stations, posters in central squares and streets of municipalities, social media, and the internet; the topics of these events were the primary and secondary prevention of breast cancer and gynecologic malignancies, as well as first aid seminars for the general public. Other activities of the project included the examination of patients residing in the border area by gynecologists, otorhinolaryngologists, and pediatricians in the project’s mobile unit, and the development of a digital information system for residents of the cross-border area regarding primary health units and emergency health cases.

The questionnaire consists of eight questions, with both closed and open answers. The first two questions are about warning signs of breast cancer, question 1 with open answers and question 2 with closed answers; question 3 is about confidence, skills and behavior, with closed answers; question 4 is about seeking help for cancer symptoms, with open answers; question 5 is about knowledge on breast cancer presentation in different age groups of women, with closed answers; question 6 is about the national breast cancer screening program in Greece, with closed answers; the last two questions are about breast cancer risk factors, question 7 with open and question 8 with closed answers.

The study inclusion criteria were the following. 1) Age 18 years and older and 2) women residing in Greece, in the rural area on the border with the Republic of North Macedonia. The study exclusion criteria were the following. 1) Communication issues, including mental disability, deafness, and/or blindness, and 2) a personal history of breast cancer.

This study was approved by the Research Committee - Special Account for Research Grants of the International Hellenic University (14th Sitting/13.11.2019, Topic 31.3). This study was funded and conducted in the frame of INTERREG IPA Cross-border Cooperation Programme “Greece - Republic of North Macedonia 2014-2020”; Project number CCI 2014 TC 16 I5CB 009; Project title: “Unified information system for exchanging information between primary health units in the cross-border area for emergency health cases”; Project acronym: HEALTH INFO.

Statistical analysis

Data were recorded using Microsoft Excel (Microsoft Corporation, Redmond, United States). Statistical analysis was performed through IBM SPSS Statistics for Windows, Version 26 (Released 2019; IBM Corp., Armonk, New York, United States). All categorical data are presented as numbers (n) and/or percentages (%) as appropriate. Continuous variables are presented as mean and standard deviation (±SD). As data were qualitative, the non-parametric tests, χ^2^ goodness-of-fit, and Cramer’s V test for categorical comparisons were used. Cronbach’s alpha was used for reliability analysis for certain questionnaire parts, as appropriate. The level of statistical significance was set as p < 0.05.

## Results

In total, 110 women filled out and returned the questionnaire. Demographic characteristics of participants are presented in Table 1. The mean age of women was 33 years (±16.67). Regarding race and ethnic background, 93% of participants were white and 93% were of Greek descent, respectively. With respect to educational background, 69.1% were students or had completed tertiary education and 25.4% had completed secondary education. Regarding the professional status of participants, 43.6% were students, 18.2% were employed in the public, 11.8% in the private sector, and 12.7% were unemployed. Subgroup analyses did not show any differences between subgroups according to different demographic characteristics, i.e., age, race, ethnic and educational background, and professional status.

**Table 1:** Demographic characteristics of participants (n = 110)

Characteristic	Mean (SD) or rate (%)
Age	33 years (±16.67)
Race	
White	93%
Other	2.6%
Preferred not to answer	4.4%
Ethnic background	
Greek	93%
Albanian	3.6%
Other	3.4%
Educational background	
Students or completed tertiary education	69.1%
Completed secondary education	25.4%
Completed primary education	4.5%
Preferred not to answer	1%
Professional status	
Students	43.6%
Employed in the public	18.2%
Employed in private sector	11.8%
Unemployed	12.7%
Self-employed	6.4%
Retired	7.3%

The first question was about warning signs of breast cancer (“Please name as many early warning signs of breast cancer as you can think of”) with open answers (Table 2). About 31.3% of women gave 1-2 correct and 38.6% gave 3-4 correct answers (p < 0.001); however, 64.3% gave 1-2 false answers (p = 0.001).

**Table 2:** Open answers to question 1, “Please name as many early warning signs of breast cancer as you can think of”

Number of answers	N	Rate %
0 correct answers	8	9.6%
1-2 correct answers	26	31.3%
3-4 correct answers	32	38.6%
5 correct answers	9	10.8%
6-10 correct answers	7	8.4%
11 correct answers	1	1.2%
0 false answers	8	28.6%
1-2 false answers	18	64.3%
2-4 false answers	2	7.1%
>5 false answers	0	0

The second question was again about warning signs of breast cancer (“Can you tell me whether you think any of these are warning signs of breast cancer or not?”) with 11 closed answers; findings are presented in Table 3. Overall, Cronbach’s alpha was 0.633 for question 2, suggesting modest to high reliability. It is noteworthy that most respondents were aware that a breast lump (94.7%) and a lump in the axilla (78.9%) are possibly warning signs of breast cancer, and that the majority (>50%) recognized six of the other breast cancer signs, i.e., nipple discharge, pulling of the nipple skin, changes in the size and shape of the nipple and/or the breast, breast pain and dimpling of the breast skin; however, more than half of respondents did not recognize that redness of the breast skin, a rash around the nipple and a change in the position of the nipple are possibly warning signs of breast cancer.

**Table 3:** Closed answers to question 2, “Can you tell me whether you think any of these are warning signs of breast cancer or not?”

Question	Answers			p-value
	Yes	No	Don’t know	
Do you think a lump or thickening in your breast could be a sign of breast cancer?	94.7%	3.2%	2.1%	p < 0.001
Do you think a lump or thickening under your armpit could be a sign of breast cancer?	78.9%	7.3%	13.8%	p < 0.001
Do you think bleeding or discharge from your nipple could be a sign of breast cancer?	66.1%	12.8%	21.1%	p < 0.001
Do you think the pulling in of your nipple could be a sign of breast cancer?	52.7%	18.2%	29.1%	p < 0.001
Do you think a change in the position of your nipple could be a sign of breast cancer?	48.6%	17.4%	33.9%	p < 0.001
Do you think a rash on or around your nipple could be a sign of breast cancer?	45.0%	35.8%	19.3%	p < 0.001
Do you think redness of your breast skin could be a sign of breast cancer?	31.8%	39.1%	29.1%	p = 0.41
Do you think a change in the size of your breast or nipple could be a sign of breast cancer?	56.8%	19.8%	23.4%	p < 0.001
Do you think a change in the shape of your breast or nipple could be a sign of breast cancer?	61.8%	11.8%	26.4%	p < 0.001
Do you think pain in one of your breasts or armpit could be a sign of breast cancer?	58.6%	25.2%	16.2%	p < 0.001
Do you think dimpling of the breast skin could be a sign of breast cancer?	53.6%	16.4%	30.0%	p < 0.001

The third question was about confidence, skills, and behavior, subdivided into three questions with closed answers; these findings are presented in Table 4. It is worth noting that most women do not regularly perform breast self-examination: 36.4% replied “rarely or never” and 30% “once every six months”. Most women answered that they were “fairly confident” in noticing a change in their breasts (52.3%) and most women answered that they had never noticed a change in their breasts (56.7%).

**Table 4:** Closed answers to question 3 regarding confidence, skills, and behavior toward breast cancer, subdivided into three questions

Question	Answers	Rate (%)	p-value
How often do you check your breasts?	Rarely or never	36.4	p = 0.002
	Once every six months	30.0	
	Once a month	21.8	
	Once a week	11.8	
Are you confident you would notice a change in your breasts?	Not at all confident	6.4	p < 0.001
	Not very confident	24.8	
	Fairly confident	52.3	
	Very confident	16.5	
Have you ever been to see a doctor about a change you have noticed in one of your breasts?	Yes	27.0	p < 0.001
	No	16.2	
	Never noticed a change in one of my breasts	56.8	

The fourth question was about seeking help for breast cancer symptoms, with open answers (“If you found a change in your breast, how soon would you contact your doctor?”); 93.1% answered that they would contact a physician within two weeks (p < 0.001).

**Figure 1:**
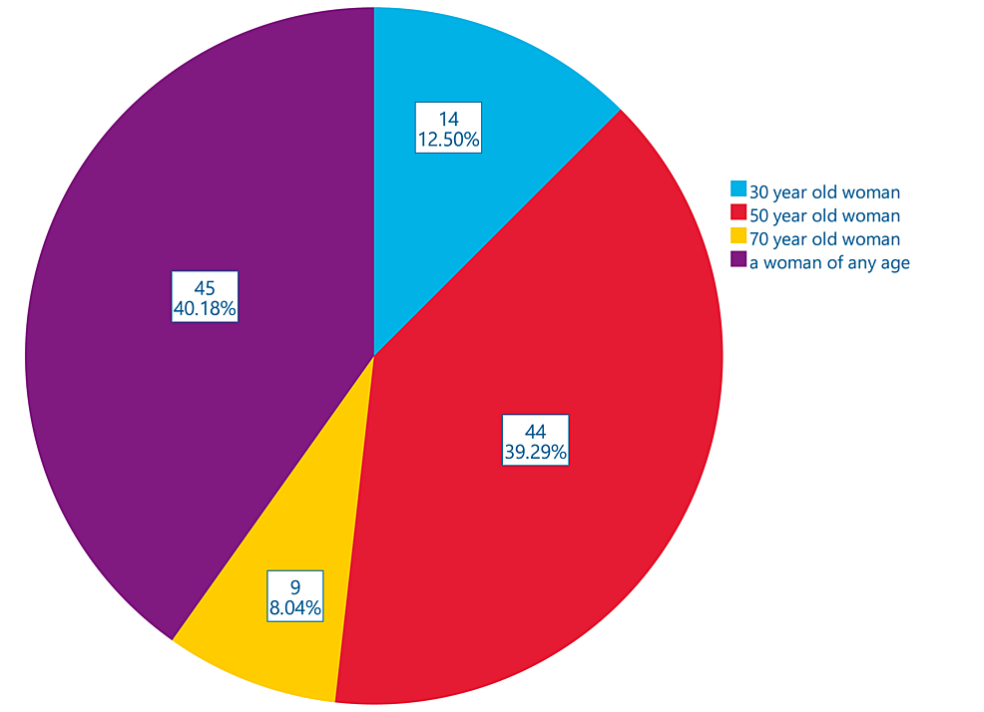
Closed answers to question 5, “In the next year, who is most likely to develop breast cancer?” Surprisingly, only 8.0% gave the correct answer, i.e., “a 70-year-old woman”

The fifth question was about knowledge of breast cancer presentation in different age groups of women (“In the next year, who is most likely to develop breast cancer?”) with four closed answers, i.e., 1) a 30-year-old woman, 2) a 50-year-old woman, 3) a 70-year-old woman and 4) a woman of any age. Unexpectedly, only 8.0% gave the correct answer, i.e., “a 70-year-old woman” (Figure 1).

The sixth question was about the national breast cancer screening program in Greece, subdivided into four questions with closed answers; these findings are presented in Table 5. Overall, Cronbach’s alpha was 0.390 for question 6, suggesting rather low reliability. Interestingly, almost half of the participants (50.5%) were not aware of the existence of a national breast cancer screening program in Greece, and only about a third (32.4%) knew the correct answer that breast cancer screening begins at 40 years of age.

**Table 5:** Closed answers to question 6 regarding the national breast cancer screening program in Greece, subdivided into four questions

Question	Answers	Rate (%)	p-value
As far as you are aware, is there a national Breast Screening Program in Greece?	No	50.5%	p = 0.92
	Yes	49.5%	
At what age may women have their first mammography in the Greek National Health Service?	20-30	15.7%	p < 0.001
	30-40	37.3%	
	40-50	32.4%	
	50-60	0.0%	
	Other	14.7%	
At what age may women have their first breast ultrasound in the Greek National Health Service?	20-30	24.2%	p = 0.053
	30-40	36.8%	
	40-50	18.9%	
	50-60	0.0%	
	Other	20.0%	
Have you ever had a breast examination? If yes, with mammography, ultrasound, or both?	No	53.3%	p < 0.001
	Yes	3.8%	
	Mammography	3.8%	
	Ultrasound	8.6%	
	Both	30.5%	

The seventh question was about breast cancer risk factors (“What things do you think affect a woman’s chance of developing breast cancer?”) with open answers (Table 6). It is noteworthy that 70.8% of participants gave 1-2 false answers.

**Table 6:** Open answers to question 7, “What things do you think affect a woman’s chance of developing breast cancer?”

Number of answers	N	Rate %
0 correct answers	3	3.3%
1-3 correct answers	51	55.4%
4 correct answers	17	18.5%
5-8 correct answers	18	19.6%
9 correct answers	3	3.3%
0 false answers	8	16.7%
1-2 false answers	34	70.8%
2-3 false answers	5	10.4%
4 false answers	1	2.1%
>5 false answers	0	0

The eighth question was again about breast cancer risk factors (“How much do you agree that each of these can increase the chance of developing breast cancer?”) with nine closed answers; overall, Cronbach’s alpha was 0.7 for question 8, suggesting high reliability; findings are presented in Table 7. It is noteworthy that most respondents knew that a personal and a family history of breast cancer are associated with an increased risk of developing breast cancer, while most participants were not aware of risk factors such as the use of hormone replacement therapy, alcohol consumption, nulliparity or primiparity in advanced age, early menarche, late menopause, and limited physical activity.

**Table 7:** Closed answers to question 8, “How much do you agree that each of these can increase the chance of developing breast cancer?”

Question	Answers					p-value
	Strongly disagree	Disagree	Not sure	Agree	Strongly agree	
Having a past history of breast cancer	0.9%	2.7%	7.1%	47.8%	37.2%	p < 0.001
Using hormone replacement therapy	3.5%	5.3%	43.4%	32.7%	8.8%	p < 0.001
Drinking more than 1 unit of alcohol a day	2.7%	33.6%	30.1%	25.7%	1.8%	p < 0.001
Being overweight (BMI over 25)	3.5%	14.2%	30.1%	37.2%	8.8%	p < 0.001
Having a close relative with breast cancer	3.5%	5.3%	8.0%	44.2%	34.5%	p < 0.001
Having children later on in life or not at all	6.2%	12.4%	39.8%	24.8%	10.6%	p < 0.001
Starting your periods at an early age	7.1%	19.5%	45.1%	11.5%	8.8%	p < 0.001
Having a late menopause	5.3%	18.6%	41.6%	19.5%	8.8%	p < 0.001
Doing less than 30 minutes of moderate physical activity five times a week	7.1%	26.5%	32.7%	23.0%	4.4%	p < 0.001

## Discussion

Evidence suggests that breast cancer patients residing in rural areas are diagnosed with more advanced diseases, most likely due to limited access to screening programs and specialist healthcare provision [9]. Cancer treatment delay is a widely recognized marker of worse outcomes, linked with an increased risk of recurrence and mortality [10,11]. Breast cancer awareness, especially the awareness of signs and symptoms of breast cancer, may prompt seeking specialist care with subsequent improvement of disease outcomes [4-6].

In the present study, we investigated the level of breast cancer awareness of women residing in a rural border area in Greece, with the aim of improving breast cancer prevention services and disease prevention policies. We used a translated modified questionnaire, based on BCAM, which was originally developed in the United Kingdom [7]. The Greek version of BCAM was handed out to women attending educational events on primary and secondary prevention of breast cancer and gynecologic malignancies, as well as first aid seminars for the general public in the frame of the “HEALTH INFO” Project [8]. Though these educational events are not yet organized on a regular basis, they could well be a part of regular and continuing activities for specific regional and population settings, such as border areas, in cooperation with local authorities and local stakeholders.

Overall, our findings suggest that women appeared to be inappropriately informed regarding the less common warning signs of breast cancer, the most common age of breast cancer occurrence, the national screening program, and the less important risk factors of breast cancer. On the other hand, most women appeared to be confident in recognizing breast changes and seeking medical advice if needed. Hence, policymakers should develop appropriate campaigns in the future to improve the level of breast awareness among women residing in rural border areas in Greece. A limitation of the present study is that since participants were residents of a certain geographical area, our findings cannot be generalized for other areas or the whole country; another limitation is that BCAM includes questions with open answers, leading to results with high variability. On the other hand, the main strengths of this study are the fact that it is original, since to the best of our knowledge there are no other studies investigating breast cancer awareness in Greece and that it is focused on a special population in a rural border area.

Translated versions of BCAM have been previously used in different countries [12-16]. In detail, the BCAM version translated to Arabic was used in Oman, with 972 Arab women participating in this study [12]; a BCAM version translated to Swahili was used in Kenya, with 48 women participating in cognitive focus group discussions, and 1,061 for surveys [13]; a version translated to Persian was used in Iran, with 1,078 participants [14]; in China, a Chinese version of BCAM was used in a study with 328 women [15]; a BCAM version translated to Malay was used in 251 Malay-speaking women across the three main ethnic groups of Malaysia, 85 Malay, 84 Chinese and 82 Indian adults [16]. In the present study, a BCAM version translated to Greek was used for the first time in 110 women residing in a rural border area in Greece; the Greek version of the questionnaire should be further evaluated in future studies in terms of reliability. In line with previous studies, it seems that different BCAM versions may be applied in different ethnic, cultural, and social settings, and healthcare professionals may use this tool to evaluate women’s knowledge about the warning signs and risk factors of breast cancer and prompt them not to delay seeking specialist care [12-16].

Besides BCAM, other tools have been used to increase and/or investigate breast cancer awareness, including small focus groups [17] and mobile applications [18]. In various studies, in different countries, including Malaysia [18], Ethiopia [19], Nigeria [20,21], and Pakistan [22] breast cancer awareness has been studied together with breast self-examination. However, breast cancer awareness is not the same and it should be distinguished from breast self-examination (BSE) [4]. BSE is a regular, repetitive palpation using a rigorous set method that must be properly performed by women at the same time each month, but level I evidence suggests that BSE has no effect on mortality; rather, BSE has potential harms, including increased psychological stress and unnecessary imaging tests and biopsies in women without cancer [4]. With breast awareness, women do not have to examine their breasts; they simply have to familiarize themselves with their breasts as a normal part of caring for their bodies [4].

## Conclusions

The translated modified BCAM tool can be used to evaluate breast awareness in Greek women. Initial findings suggest that women residing in rural border areas in Greece are not appropriately informed regarding the warning signs, the most common age of breast cancer occurrence, the national screening program, and the less important risk factors of breast cancer, while most women appeared to be confident in recognizing breast changes and seeking medical advice if needed. Given that breast cancer awareness is linked with disease outcomes, future campaigns developed by policymakers should focus on improving breast awareness, especially in socioeconomically disadvantaged areas.

## Appendices

Questionnaire for the evaluation of breast cancer awareness in Greece

1. Please name as many early warning signs of breast cancer as you can think of (complaints, symptoms, etc.)

2. Can you tell me whether you think any of these are warning signs of breast cancer or not?

**Table 8:** Question 2

Do you think a lump or thickening in your breast could be a sign of breast cancer?	YES	NO	DON’T KNOW
Do you think a lump or thickening under your armpit could be a sign of breast cancer?			
Do you think bleeding or discharge from your nipple could be a sign of breast cancer?			
Do you think the pulling in of your nipple could be a sign of breast cancer?			
Do you think a change in the position of your nipple could be a sign of breast cancer?			
Do you think a rash on or around your nipple could be a sign of breast cancer?			
Do you think redness of your breast skin could be a sign of breast cancer?			
Do you think a change in the size of your breast or nipple could be a sign of breast cancer?			
Do you think a change in the shape of your breast or nipple could be a sign of breast cancer?			
Do you think pain in one of your breasts or armpit could be a sign of breast cancer?			
Do you think dimpling of the breast skin could be a sign of breast cancer?			

3. Breast self-examination

**Table 9:** Question 3

How often do you check your breasts? (breast self-examination)	Rarely or never	once every 6 months	once a month	once a week
	Are you confident you would notice a change in your breasts?	Not at all confident	Not very confident	Fairly confident	Very confident
Have you ever been to see a doctor about a change you have noticed in one of your breasts?	YES	NO	Never noticed a change in one of my breasts	

4. Seeking medical help. If you found a change in your breast, how soon would you contact your doctor?

5. Breast cancer and age

**Table 10:** Question 5

In the next year, who is most likely to develop breast cancer?, 2) A 50 year old woman, 3) A 70 year old woman and 4) A woman of any age.;		
A 30 year old woman		
A 50 year old woman		
A 70 year old woman		
A woman of any age		

6. National breast cancer screening program in Greece

**Table 11:** Question 6

As far as you are aware, is there a national Breast Screening Program in Greece?	
At what age may women have their first mammography in the Greek National Health Service?	
At what age may women have their first breast ultrasound in the Greek National Health Service?	
Have you ever had a breast examination? If yes, with mammography, ultrasound or both?	

7. Risk factors for developing breast cancer

What things do you think affect a woman’s chance of developing breast cancer?

Name all risk factors you can think of.

8. How much do you agree that each of these can increase the chance of developing breast cancer?

**Table 12:** Question 8

	Strongly disagree	Disagree	Not sure	Agree	Strongly agree
Having a past history of breast cancer					
Using HRT (Hormone Replacement Therapy)					
Drinking more than 1 unit of alcohol a day					
Being overweight (BMI over 25)					
Having a close relative with breast cancer					
Having children later on in life or not at all					
Early menarche (Starting your periods at an early age)					
Having a late menopause					
Doing less than 30 mins of moderate physical activity 5 times a week					
